# Navigating Hurdles: A Review of the Obstacles Facing the Development of the Pandemic Treaty

**DOI:** 10.1007/s44197-024-00233-5

**Published:** 2024-04-29

**Authors:** Haytham A. Sheerah, Shouq M. Alzaaqi, Ahmed Arafa, Shada AlSalamah, Nelly G. Muriungi, Barbara Fialho C Sampaio, Jasper Tromp, Keyang Liu, Kokoro Shirai, Mellissa Withers, Ahmed Al-Jedai

**Affiliations:** 1 Office of the Vice Minister of Health, Ministry of Health, Riyadh, Saudi Arabia; 2 Immunology Research Program, King Abdullah International Medical Research Center, Riyadh, Saudi Arabia; 3 King Saud bin Abdulaziz University for Health Sciences, Riyadh, Saudi Arabia; 4 Department of Preventive Cardiology, National Cerebral and Cardiovascular Center, Suita, Japan; 5 Department of Public Health and Community Medicine, Faculty of Medicine, Beni-Suef University, Beni-Suef, Egypt; 6 Information Systems Department, College of Computer and Information Sciences, King Saud University, Riyadh, Saudi Arabia; 7 Royal (Dick) School of Veterinary Studies, University of Edinburgh, Edinburgh, Scotland; 8 Pathology Department, School of Medicine, Universidade de São Paulo, São Paulo, Brazil; 9 InCor, Hospital das Clinicas, School of Medicine, Universidade de São Paulo, São Paulo, Brazil; 10 Saw Swee Hock School of Public Health, National University of Singapore & National University Health System, Singapore, Singapore; 11 Duke-NUS Medical School, Singapore, Singapore; 12 Department of Social Medicine, Public Health, Osaka University Graduate School of Medicine, Osaka, Japan; 13 Department of Population and Health Sciences, Institute for Global Health, University of Southern California, Los Angeles, CA USA; 14 Therapeutic Affairs, Ministry of Health, Riyadh, Saudi Arabia; 15 Colleges of Medicine and Pharmacy, Alfaisal University, Riyadh, Saudi Arabia

**Keywords:** COVID-19, Pandemic treaty, Healthcare, Global health, Public health

## Abstract

**Introduction:**

The emergence of the COVID-19 pandemic has served as a call for enhanced global cooperation and a more robust pandemic preparedness and response framework. As a result of this pressing demand, dialogues were initiated to establish a pandemic treaty designed to foster a synchronized global strategy for addressing forthcoming health emergencies. In this review, we discussed the main obstacles to this treaty.

**Results:**

Among several challenges facing the pandemic treaty, we highlighted (1) global cooperation and political will, (2) equity in access to resources and treatments, (3) sustainable financing, (4) compliance and enforcement mechanisms, (5) sovereignty concerns, and (6) data sharing and transparency.

**Conclusion:**

Navigating the hurdles facing the development of the pandemic treaty requires concerted efforts, diplomatic finesse, and a shared commitment to global solidarity. Addressing challenges in global cooperation, equitable access, transparency, compliance, financing, and sovereignty is essential for forging a comprehensive and effective framework for pandemic preparedness and response on the global stage.

## Introduction

In the wake of the SARS pandemic in 2005, 196 countries across the globe have agreed to implement the International Health Regulations (IHR), a legally binding instrument of international law developed by the World Health Organization (WHO) as a neutral agency aiming to help the international community prevent and respond to acute public health risks [1]. The key components of the IHR, which came into force in 2007, involved (1) core capacities, including surveillance and reporting systems, laboratory capabilities, and public health infrastructure, (2) notification and verification, (3) coordination and collaboration, (4) travel and trade measures, and (5) monitoring and evaluation [1]. However, the IHR failed to efficiently respond to the challenges brought by the COVID-19 pandemic [2–4]. This inefficient response was mainly attributed to the lack of political commitment, limited enforcement mechanisms, unequal capacity and resources, geopolitical issues, fragmentation of global health governance, and misinformation [2–4].

The significant shortcomings in the global response to COVID-19 have underscored the urgent need for formulating a new method of enforcing global cooperation in pandemic preparedness and response. In response to this pressing need, discussions about a pandemic treaty have gained traction in international forums [5]. Such a treaty is supposed to bolster global cooperation, improve coordination, and enhance transparency in dealing with pandemics in a way that avoids the IHR limitations [5].

On the 30th of March 2021, 25 heads of government and international agencies came together in an extraordinary joint call for an international pandemic treaty [6]. Later, more governments and agencies expressed their interest in that treaty [7]. On the 1st of December 2021, the 194 member states of the WHO agreed to begin negotiations towards achieving the pandemic treaty [8]. On the 1st of February 2023, the Intergovernmental Negotiating Body (INB), tasked with drafting and negotiating the pandemic treaty, released a zero draft for the consideration of WHO member states [9]. The proposed treaty adheres to the principles of equity, human rights, and unity, acknowledging the sovereign rights of nations, disparities in development levels, and the pertinent international agreements in place [10]. It offers numerous potential advantages, particularly for low- and middle-income countries (LMICs). These advantages include improving access to essential pandemic-related products and technologies, prioritizing health system resilience in preparedness efforts, broadening fair access to and involvement in global sharing of pathogen and genomic sequencing data, advocating for comprehensive pandemic literacy initiatives involving all sectors of society, incorporating concerns about climate change and environmental deterioration, and strengthening representation and engagement in global health governance [10]. To avoid overlap, states are deliberating the suggested revisions to the International Health Regulations (IHR, 2005), taking into account the hurdles presented by the COVID-19 pandemic, with guidance from the Working Group on Amendments to the IHR (WGIHR) [3].

However, the road to drafting and implementing such a treaty is fraught with numerous challenges related to the complexities of diplomatic negotiations, legal frameworks, and resource allocation. Moreover, the development of the pandemic treaty intersects with broader issues of global health governance, equity, and ethical considerations. From disparities in access to healthcare resources to questions of sovereignty and human rights, the treaty’s formulation demands scrutiny of its legal and ethical implications. Herein, we discuss the challenges facing the development and implementation of the pandemic treaty. Elucidating these multifaceted obstacles not only serves to enhance our understanding of the complexities inherent in global health diplomacy but also informs strategic interventions necessary for a resilient and equitable global health landscape.

## Methods

We addressed the obstacles facing the development of the pandemic treaty using a narrative review methodology, which utilizes existing evidence from published literature and reports. This approach serves as a connection between established literature and contemporary events, providing critical analysis.

## The Challenges of the Pandemic Treaty

### Global Cooperation and Political will

One of the foremost challenges in establishing the pandemic treaty was garnering widespread global cooperation and political will. Every member of the European Union has endorsed the pandemic treaty, which is also backed by the African Union, Asian, and South American governments [11]. Yet, despite the large number of governments and agencies involved in the negotiations, it should be noted that several stakeholders advocating for the pandemic treaty negotiations, especially high-income countries (HICs), stockpiled vaccines and opposed an intellectual property (IP) waiver initiative introduced by India and South Africa in 2020 during the COVID-19 pandemic, a stance they maintained for two years [12, 13]. Numerous governments veered away from the WHO guidelines to contain COVID-19 spread, discarded the rhetoric of global unity, and restricted the export of medical resources [12, 13]. Evaborhene and colleagues criticized the zero draft of the pandemic treaty for not including clear incentives and disincentives for political leaders, prompting them to alter their behavior in future outbreaks [13]. Thus, we ought to approach this issue with skepticism as to why these stakeholders might exhibit varying responses in future outbreaks.

### Equity in Access to Resources and Treatments

Establishing institutional frameworks for the pandemic treaty should be based on fundamental human rights principles. Key principles such as universal healthcare access, the pivotal function of public health infrastructure, and the guarantee of substantive equality to address diverse human needs should constitute the foundational pillars of human rights concerning health [14]. Yet, ensuring equitable access to vaccines, treatments, and healthcare services and resources is another significant challenge [4].

The COVID-19 pandemic has exposed stark disparities in access to essential medical supplies and healthcare services, within and between countries. A systematic review of 22 articles (15 from the US, 3 from Europe, 3 from Asia, and 1 from Oceania) concluded that COVID-19 vaccine distribution was significantly influenced by economic, legal, logistic, epidemiologic, and demographic factors [15]. The lack of technology transfer from vaccine producers in HICs to manufacturers in LMICs has presented a significant obstacle to the swift expansion of the global COVID-19 vaccine supply [4]. In Africa, for example, due to the lack of a widely distributed vaccine manufacturing infrastructure, African nations were compelled to depend on the COVID-19 vaccine global access initiative (COVAX), which pledged to ensure equitable vaccine access regardless of a country’s economic status. Nevertheless, several HICs, hoarding doses beyond their population requirements, sidelined the COVAX initiative, relegating it to a lower priority among buyers [13]. Thus, the pandemic treaty must address these inequities by promoting fair distribution mechanisms and facilitating technology transfer to enhance local manufacturing capacities.

While the zero draft of the pandemic treaty has indicated that the WHO would receive 20% of pandemic-related products, half of them would be provided free of cost and the remaining half would be provided at affordable prices [9]. Evaborhene and colleagues expressed their skepticism that implementation would mainly depend on negotiations [16].

According to Perehudoff and colleagues, the pandemic treaty has to mandate technology transfer [4]. To accomplish this goal, they suggested establishing two categories of obligations for governments. Firstly, governments should tie public research and development funding for medical countermeasures to agreements ensuring substantial technology transfer. Secondly, governments should collaborate to implement mandates, subsidies, and incentives for the private sector to participate in technology transfer to eligible entities, irrespective of whether such knowledge is funded by the public sector [4].

### Sustainable Financing

Adequate financing is crucial for building resilient health systems, conducting research, and implementing pandemic preparedness measures. However, securing sustainable funding for pandemic response efforts poses a challenge, particularly in the face of competing global priorities and economic uncertainties. There is a prevailing understanding that private investments in vaccines and treatments for potential pandemics are insufficient, particularly in the pre-pandemic phase [4]. Therefore, initiating policies for accessing efficient medical countermeasures should involve government-led initiatives directly or indirectly financing, subsidizing, incentivizing, and mitigating risks in their development. Innovative financing mechanisms, such as pandemic bonds and solidarity levies, may offer viable solutions to bridge the funding gap [4].

The World Bank Pandemic Fund could bring additional resources for pandemic prevention, preparedness, and response, incentivize countries to increase investments, enhance coordination among partners, and serve as a platform for advocacy. In response to the COVID-19 pandemic, the World Bank financial intermediary funds exceeded 1.2 billion USD. The European Union (465 million USD), the United States (450 million USD), Germany (122 million USD), Italy (106 million USD), Japan (70 million USD), France (54 million USD), China (50 million USD), Saudi Arabia (50 million USD), and Indonesia (50 million USD) provided most of these funds which were devoted to several projects related to the COVID-19 pandemic prevention, preparedness, and response activities [17]. We suggest the pandemic treaty should consider increasing the World Bank Pandemic Fund. This increase can enhance global readiness and resilience in the face of outbreaks, ultimately contributing to better pandemic control and management worldwide. The increased funds may also allow quicker and more effective responses to outbreaks, allocating healthcare infrastructure, medical supplies, vaccines, and research, and helping LMICs strengthen their healthcare systems and response capacities.

### Compliance and Enforcement Mechanisms

Even with the establishment of the pandemic treaty, ensuring compliance with its provisions remains a formidable challenge. Unlike traditional treaties with clear enforcement mechanisms, enforcing compliance in the context of the pandemic treaty poses unique difficulties. For instance, despite the clear legal obligations outlined in the IHR, several countries did not comply with all requirements [2–4]. Therefore, Faviero and colleagues called for accountability mechanisms including a robust system of incentives and disincentives, such as binding countries’ compliance with preparedness and response regulations to the International Monetary Fund (IMF) periodic evaluation of their economic indices [18]. Evaborhene and colleagues highlighted the challenges in understanding how pharmaceutical companies, reluctant to share technologies and prioritize shareholders’ dividends, would reconcile corporate social responsibility with profit-making when fulfilling the non-donated 10% outlined in the zero draft [13, 16]. Jiang and Kumah argued that the current draft may inadvertently sustain health disparities rather than alleviate them due to the lack of a strong mechanism to tackle non-compliance and settle disputes, coupled with the absence of specific incentives or penalties to encourage adherence. LMICs might face challenges in meeting the expectations and obligations outlined in the treaty. This could endanger their access to crucial resources and assistance during health crises [11]. Therefore, we believe that the vagueness of compliance and enforcement mechanisms is a major limitation in the pandemic treaty zero draft.

### Balancing National Sovereignty and Global Solidarity

National sovereignty implies that states have the authority to govern their affairs without external interference, including in matters of public health. However, in the context of a global health crisis, such as a pandemic, actions taken by one nation can have significant repercussions beyond its borders [19, 20]. A tension between national sovereignty and global solidarity may arise when countries seek to protect their sovereignty by prioritizing domestic interests, such as securing vaccine supplies or implementing travel restrictions, which may impede efforts to achieve collective goals, such as equitable access to vaccines and coordinated pandemic response measures [19, 20].

Promoting global solidarity requires countries to collaborate and pool resources to address common challenges collectively. This may involve sharing data, coordinating public health measures, and distributing essential medical supplies across borders. However, concerns about sovereignty can hinder cooperation, as nations may be reluctant to cede control over their resources or accept international oversight. Additionally, disparities in wealth, healthcare infrastructure, and political priorities further complicate efforts to promote solidarity, as some countries may perceive themselves as bearing a disproportionate burden in supporting LMICs [19, 20].

Furthermore, one of the conjectures regarding the pandemic treaty suggests it could grant the WHO an unlimited mandate to determine the status and measures for pandemic control and response. This would entail the WHO imposing strict restrictions and penalties on the use of medicines and diagnostics prohibited by the WHO, even if proven effective in local contexts. Additionally, it might involve implementing lockdowns, overriding national public health laws, imposing travel restrictions, and censorship, thereby limiting the autonomy of countries and individuals [21–23].

Despite the tension between upholding national sovereignty and promoting global solidarity, global health security should prioritize the security of people rather than national borders [14, 19]. The rapid spread of COVID-19 and its variants has proved that putting national interest above mutual global action was not only immoral but complicated and weakened the global response as well [14, 19]. Therefore, we believe that the pandemic treaty should on the one hand outline provisions that safeguard national sovereignty by respecting the autonomy of each member state in making decisions related to public health and on the other hand implement shared decision-making mechanisms within the treaty framework to enable collaborative decision-making among member states.

### Data Sharing and Transparency

An effective pandemic response relies on the timely and transparent sharing of data and information that would facilitate making informed decisions. To achieve that, data should include outbreak metrics, such as incidence, mortality, number of people tested, test positive rate, number of patients hospitalized, number of patients discharged, the proportion of the population who received at least one vaccine, and the proportion of the population fully vaccinated. These metrics should be stratified by age, sex, ethnicity/race, and socio-economic status. Data on subgroups, such as residents in nursing homes, inmates, students, healthcare and social workers, and residents in refugee camps, should also be available [24].

To solve this issue, a recent meeting of the INB has proposed the creation of a multilateral access and benefit-sharing system for pathogens with pandemic potential, known as the “WHO Pathogen Access and Benefit Sharing System” (PABS System). This system aims to guarantee swift, organized, and timely sharing of PABS material and information for public health risk assessment. Additionally, it aims to ensure fair and equitable access to pandemic-related health products, as well as other benefits, whether monetary or non-monetary, resulting from such sharing [25].

As per Hampton and colleagues, ABS has failed to produce fair results in international environmental law over the past three decades. They suggested that if member states sincerely aim to achieve outcomes resembling equity in future pandemics, they should prioritize regional capacity building, technology, and knowledge transfer beforehand. Incorporating these elements through ABS in the pandemic treaty may create counterproductive incentives and foster adversarial relationships, rather than the collaboration and solidarity required [26]. Moreover, concerns about data privacy, intellectual property rights, and national security also can hinder the sharing of critical information during health emergencies [21].

## Conclusion

The challenges facing the development and implementation of the pandemic treaty are multifaceted and require concerted efforts from the international community. These challenges include global cooperation and political will, equity in access to resources and treatments, sustainable financing, compliance and enforcement mechanisms, sovereignty concerns, and data sharing and transparency are the main obstacles facing the development of the pandemic treaty. Despite these challenges, the urgency of addressing global health threats demands bold and collaborative action.

To achieve global cooperation, a shared recognition of the common threat of pandemics is required. Securing agreement on pivotal matters, such as vaccine allocation, surveillance methods, and financing, is imperative to ensure equity in access to resources and treatments. Enhancing compliance with the pandemic treaty requires efficient peer monitoring and review mechanisms. Additionally, striking a balance between respecting national autonomy and fostering international cooperation is essential for the success of the pandemic treaty. Finally, developing mechanisms to promote data sharing while safeguarding privacy is a complex task that requires careful deliberation.

## Data Availability

No datasets were generated or analysed during the current study.

## Abbreviations

COVAX: COVID-19 vaccine global access initiative
HIC: High-income countries
IHR: International Health Regulations
IMF: International Monetary Fund
INB: Intergovernmental Negotiating Body
IP: Intellectual property
LMIC: Low- and middle-income countries
WGIHR: Working Group on Amendments to the International Health Regulations
WHO: World Health Organization

## Declarations

The authors confirm that their governmental positions or membership in relevant committees did not influence the research outcomes. This paper and the opinions stated herein do not represent the official stand of member states of the authors.
